# Fibroblast growth factor-23 is a strong predictor of insulin resistance among chronic kidney disease patients

**DOI:** 10.1080/0886022X.2018.1455594

**Published:** 2018-04-05

**Authors:** Ahmed Fayed, Mahmoud M. El Nokeety, Ahmed A. Heikal, Dina O. Abdulazim, Mervat M. Naguib

**Affiliations:** a Nephrology Unit, Internal Medicine Department, School of Medicine, Cairo University, Cairo, Egypt;; b Internal Medicine Department, School of Medicine, Cairo University, Cairo, Egypt;; c Rheumatology and Rehabilitation Department, School of Medicine, Cairo University, Cairo, Egypt;; d Endocrinology Unit, Internal Medicine Department, School of Medicine, Cairo University, Cairo, Egypt

**Keywords:** FGF23, CKD-MBD, insulin resistance, HOMA-IR, vitamin D, 25 OH vitamin D

## Abstract

Insulin resistance (IR) is very common among chronic kidney disease (CKD) patients. Disturbance in mineral and bone metabolism (MBD) seems to play a role in the pathogenesis of insulin resistance. Fibroblast growth factor-23 (FGF23) is evolving as the most important link between MBD and many pathologic sequences of CKD. The aim was to evaluate IR in pre-dialysis CKD patients looking for a possible association to mineral metabolism among CKD patients. A total of 100 stage 3–5 CKD patients were selected beside 20 normal control subjects. Homeostatic model assessment of insulin resistance (HOMA-IR) was used to assess IR in selected cases. Both groups were compared for fasting blood glucose (FBG), fasting blood insulin (FBI), HOMA-IR, estimated glomerular filtration rate (eGFR), serum calcium (Ca), phosphorus (P), 25 hydroxy vitamin D (25 OH vit D), parathormone (PTH), and uric acid (UA). Correlation study between HOMA_IR and different studied parameters was performed. HOMA-IR is significantly higher in CKD (8.87 ± 3.48 vs. 3.97 ± 0.34 in CKD vs. control, respectively, *p* < .001). In addition CKD patients have significantly higher FGF23 (235 ± 22.96 vs. 139 ± 12.3 pg/mL, *p* < .001), PTH (76.9 ± 15.27 vs. 47.9 ± 2.52 pg/mL, *p* < .001), P (4.3 ± 0.67 vs. 3.6 ± 0.23 mg/dL, *p* < .001), and UA (5 ± 1.22 vs. 4.85 ± 0.48 mg/dL, *p* < .001) and significantly lower Ca (8.2 ± 0.3 vs. 8.9 ± 0.33 mg/dL, *p* < .001), and 25 (OH) vit D (17 ± 5.63 vs. 37 ± 3.43 ng/mL, *p* < .001). Stepwise linear regression analysis revealed that BMI, GFR, Ca, P, and FGF23 were the only significant predictors of HOMA IR. Increased IR in CKD is a consequence of the uremic status and is intimately associated with disturbed phosphate metabolism and FGF23. Further studies are needed to look for an underlying mechanism.

## Introduction

IR is a metabolic alteration that occurs very early in CKD patients, becomes more apparent with the progress of the disease to become almost universal when CKD patients approach end-stage renal disease [1]. IR is defined as a decreased sensitivity of target organs to circulating insulin. As a consequence, pancreatic β-cells increase insulin synthesis and secretion leading to hyperinsulinemia. In CKD patients, IR is closely associated with endothelial dysfunction [2], oxidative stress and chronic inflammation [3]. IR is incriminated in cardiovascular disease in CKD patients [4] and may accelerate CKD progression [5]. These findings should pay the attention toward IR as a therapeutic target in an attempt to improve outcomes in CKD. Additional factors have been identified as significant contributors to CKD-associated IR. These factors include increased visceral fat, retention of nitrogenous compounds, metabolic acidosis, vitamin D deficiency, anemia, and physical inactivity [6]. In an older study, Ca × P product and iPTH were recognized as significant predictors of IR in non-diabetic pre-dialysis CKD [7]. Three years ago, a potential link between IR and P homeostasis was suggested in a study of seventy-two stage 3–5 CKD patients. In this study, insulin resistance significantly and independently correlated with serum carboxyl-terminal (C-terminal) FGF-23 levels [8].

FGF23 is a member of a large family of growth factors. Its’ main function is to regulate the serum level of P [9]. FGF23 is significantly associated with vascular calcification [10], inflammation [11], left ventricular hypertrophy [12], progression of renal disease [13], and secondary hyperparathyroidism [14] among CKD patients. Most of the old clinical studies on serum FGF23 concentrations have used C-terminal assays. These assays detect both intact FGF23 and its inactive C-terminal fragments. Intact FGF23 assays are now available. These assays detect the biologically active intact FGF23 exclusively [15]. The performance of C-terminal assays is discrepant [16] and these assays can produce completely inconsistent results in some particular cases [17]. As a result, readings obtained by one assay may not be always reproducible by other assays [18].

In this study, we tried to evaluate IR in pre-dialysis non-diabetic pre-dialysis CKD patients that have well-recognized cause for their renal disease. We looked for the possible association of IR to intact FGF23, serum UA, serum Ca, P, 25 OH vit D, and PTH levels in addition to eGFR and urine albumin/creatinine ratio (ACR).

## Material and methods

This study included 100 CKD cases (57 male and 43 female) and 20 normal control subjects (11 male and 9 female). In order to avoid the impact of some of the underlying causes of CKD on IR, diabetic, and hypertensive patients were excluded, besides patients with body mass index (BMI) >28 kg/m^2^, those previously or still treated with steroids, other immunosuppressive agents, vitamin D, or any phosphate binder. For the same purpose, patients with unknown cause of CKD were excluded. Most of the studied cases were in stage 4 (eGFR =15–29 mL/min/1.73 m^2^, 78 cases), while 20 cases were in stage 3 (eGFR = 30–59 mL/min/1.73 m^2^) and only two cases were in stage 5 (eGFR <15 mL/min/1.73 m^2^) [19]. Underlying etiology of renal disease in patient group is summarized in Table 1.

**Table 1.** Etiology of CKD.

Etiology	No. (%)	Etiology	No. (%)
Ch. interstitial nephritis	48 (48)	Ch. glomerulonephritis	8 (8)
Obstructive uropathy	17 (17)	Polycystic kidney	12 (12)
Reflux nephropathy	8 (8)	Hereditary nephropathy	2 (2)
Chronic gouty nephritis	5 (5)		

Estimation of IR was done using HOMA-IR which was calculated as fasting glucose (mmol/L)×fasting insulin (µU/mL)/22.5 [20]. Serum level of intact FGF23 was determined using a two-site (NH2-terminal/C-terminal) enzyme-linked immunosorbent assay (Immutopics, San Clemente, CA). According to the instructions of the manufacturer, samples were collected in the morning after 12 h fasting. The collected samples were centrifuged and the plasma was separated from the cells. Samples were assayed immediately or stored at −70 °C or below.

Intact PTH levels were determined by enzyme-amplified sensitivity immunoassay (Roche Diagnostics, Indianapolis, IN). Then, 25 OH vit D was assessed Using HPLC [21]. eGFR was measured using MDRD equation [22].

Microsoft computer statistics package (SPSS Inc., Chicago, IL) was used for data analysis. Data were summarized as a mean and standard deviation. Comparison between groups was evaluated using Mann–Whitney test. Spearman’s rank correlation coefficient between HOMA IR and different variables studied was calculated. A backward stepwise linear regression model was conducted to explore predictors of HOMA (IR) after logarithmic transformation of dependent and all independent variables.

## Results

**Figure 1.**
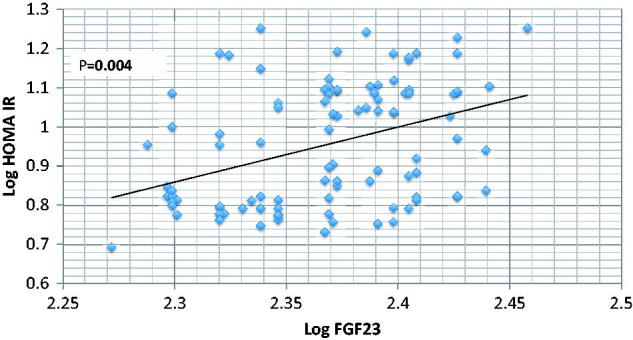
Correlation between Log FGF23 and Log HOMA IR.

**Figure 2.**
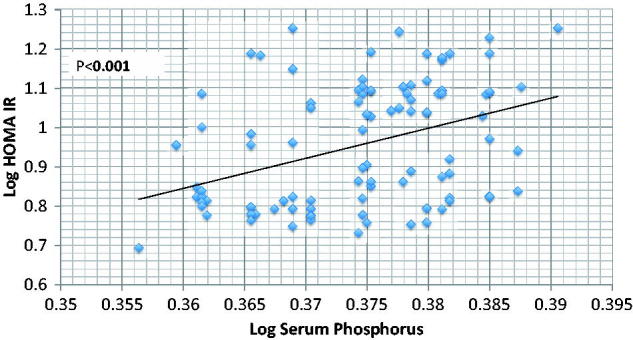
Correlation between Log serum phosphorus and Log HOMA IR.

Results are summarized in Tables 1–4 and Figures 1 and 2. Fasting insulin level is significantly higher in CKD group compared to normal control (38 ± 15.55 vs. 18 ± 1.07 mU/L in CKD vs. control respectively, *p* < .001) in spite of absence of significant difference in fasting blood glucose between the two groups (90 ± 5.36 vs. 90 ± 4.73 mg/dL in CKD vs. control, respectively, *p* > .05). HOMA-IR is significantly higher in CKD (8.87 ± 3.48 vs. 3.97 ± 0.34 in CKD vs. control respectively, *p* < .001) (Table 2). By using Spearman’s correlation rank, the only studied parameters that show significant correlation with HOMA-IR are serum P and FGF23, all other studied parameters including age, BMI, serum UA, Ca, PTH, 25 OH vit D, ACR, or eGFR failed to show similar significant correlations (Table 3). A backward stepwise linear regression model was conducted to explore predictors of HOMA (IR) after logarithmic transformation of dependent and all independent variables. All quantitative variables entered the correlation were included in the 1st step of the model. The last step revealed that only BMI, GFR, Ca, P, and FGF23 were the only significant predictors of HOMA IR (Table 4).

**Table 2.** Patients vs. control group.

	Group		
	CKD cases (*n* = 100)	Controls (*n* = 20)	*p* value
AgeMean ± SD	28.3 ± 7.5	29.2 ± 4.9	.504*
Median (IQR)	27 (22.5–33)	30 (25.5–33)	–
GenderMale	57 (57)	13 (65)	.508#
* *Female	43 (43)	7 (35)	–
Smokers	25 (25)	7 (35)	–
Non-smokers	75 (75)	13 (65)	.356#
BMI (kg/m^2^)Mean ± SD	23.2 ± 2.7	24.1 ± 2.6	–
Median (IQR)	23.5 (22–25)	25 (22.6–25.6)	.176*
BUN (mg/dL)Mean ± SD	18.5 ± 3.3	13.1 ± 2.4	<.001*
Median (IQR)	18 (16–21)	13 (11–15)	–
Creatinine (mg/dL)Mean ± SD	3 ± 0.5	0.8 ± 0.1	<.001*
Median (IQR)	3 (2.6–3.4)	0.8 (0.7–0.8)	–** **
e-GFR (mL/min/1.73 m²)Mean ± SD	24.4 ± 6	110.3 ± 31	<.001*
Median (IQR)	23.6 (19.9–28.3)	107.2 (84.8–136.6)	**–**
Ca (mg/dL)Mean ± SD	8.1 ± 0.3	9 ± 0.3	<.001*
Median (IQR)	8.2 (7.9–8.4)	8.9 (8.7–9.3)	–
Phosphorus (mg/dL)Mean ± SD	4.4 ± 0.7	3.7 ± 0.2	<.001*
Median (IQR)	4.3 (4–5.2)	3.6 (3.5–4)	–
PTH (pg/mL)Mean ± SD	75.7 ± 15.3	47.9 ± 2.5	<.001*
Median (IQR)	76.9 (64–89.4)	47.9 (46–50.4)	–
25 (OH) Vit. D (ng/mL)Mean ± SD	16.8 ± 6.5	36.6 ± 3.4	<.001*
Median (IQR)	17 (10.7–22)	37 (33.4–39.4)	–
ACR in urine (mg/g)Mean ± SD	27.7 ± 20.1	–	–
Median (IQR)	22.5 (8.7–44.8)	–	–
Fasting insulin level (mU/L)Mean ± SD	43.6 ± 15.6	18.2 ± 1.1	<.001*
Median (IQR)	38 (29.5–54)	18 (17.4–19)	–
Fasting glucose level (mg/dL)Mean ± SD	88.8 ± 5.4	88.6 ± 4.7	.710*
Median (IQR)	90 (84–93)	90 (84–93)	–
HOMA test IRMean ± SD	9.6 ± 3.5	4 ± 0.3	<.001*
Median (IQR)	8.9 (6.5–12.2)	4 (3.7–4.2)	–
FGF23 level (pg/mL)Mean ± SD	234.1 ± 23	142.3 ± 12.3	<.001*
Median (IQR)	235 (217–251.5)	139 (134–150)	–
Uric acid (mg/dL)Mean ± SD	5.4 ± 1.2	4.7 ± 0.5	<.001*
Median (IQR)	5 (4.5–6.8)	4.9 (4.3–5.2)	** **–

* Mann–Whitney test.

# Chi-square test.

eGFR: estimated glomerular filtration rate; FGF23: fibroblast growth factor-23; IR: insulin resistance; ACR: albumin/creatinine ratio.

*p* values <.05 are significant.

*p* values <.001 are highly significant.

**Table 3.** Correlation of HOMA IR with different studied variables in CKD.

	HOMA test IR	
	*r*	*p* value
Age	0.010	.920
BMI	0.051	.617
e-GFR (mL/min/1.73 m²)	−0.032	.749
Ca	−0.175	.082
PO4	**0.296**	**.003**
PTH (pg/mL)	−0.053	.598
25 (OH) Vit. D (ng/mL)	0.047	.644
Alb/cr in urine	0.020	.843
FGF23 level	**0.383**	**<.001**
Uric acid	**−0.034**	**.733**

*r*: Spearman correlation coefficient; IR: insulin resistance; BMI: body mass index; eGFR: estimated glomerular filtration rate; Ca: serum calcium; PO4: serum phosphorus; PTH: serum parathormone; Alb/cr: albumin/creatinine ratio; FGF23: fibroblast growth factor-23.

*p* values <.05 are significant.

*p* values <.001 are highly significant.

**Table 4.** Multivariate regression analysis looking for predictors to HOMA-IR in CKD patients.

	*β*	95% CI of β			*p* value
Log_BMI	−0.074	−0.147 to −0.001			**.048**
Log_GFR	−0.064	−0.118 to −0.011			**.020**
Log_Ca	−1.176	−1.430 to −0.923			**<.001**
Log_PO4	−0.208	−0.287 to −0.130			**<.001**
Log_FGF23	−0.140	−0.235 to −0.045			**.004**

β: beta coefficient; CI: confidence interval; Log: logarithmic transformation; IR: insulin resistance; BMI: body mass index; Alb: serum albumin; eGFR: estimated glomerular filtration rate; Ca: serum calcium; PO4: serum phosphorus; FGF23: fibroblast growth factor-23.

*p* values <.05 are significant.

*p* values <.001 are highly significant.

## Discussion

Increased IR is a universal metabolic feature that proved as an independent predictor of mortality in early as well as late stages of CKD [4,23]. It is not clear if the IR in CKD is a consequence of the uremic status or is secondary to the underlying cause of CKD. In order to confirm if increased insulin resistance among CKD patients is related to the uremic milieu, we tried to exclude patients that have underlying diseases with an established increase in IR. In most of the adult communities worldwide, diabetes mellitus (DM) and systemic hypertension are responsible for more than two-thirds of CKD cases. While increased IR is a common feature of type 2 DM, essential hypertension is considered an IR state [24,25]. Similarly, obese patients and those kept on steroid treatment or other immunosuppressive medications could have increased IR. Based on these facts, we have excluded diabetic, hypertensive, and obese patients in this study besides those already treated or have a history of steroid or other immunosuppressive treatment. These restrictions have posed a tough burden on the investigators while selecting the CKD patients recruited for this study. In spite of all these considerations, HOMA-IR was found significantly increased in comparison to the normal control subjects. This finding confirms that increased IR in CKD is a consequence of the uremic status.

During January 2014, Garland and her colleagues announced for the first time the strong association between IR and FGF23 in type 2 DM patients suffering CKD. FGF 23 was measured in that study using c-terminal (ctFGF-23) assay. In CKD patients, this assay measures both the inactive c-terminals beside the biologically active intact molecule, rendering assessment difficult to interpret. They concluded that increasing level of HOMA-IR was a risk factor for rising ctFGF-23 levels [8]. This conclusion seems improper. If IR stimulates FGF23, this increase of IR should have resulted in a negative correlation between IR and serum P. FGF23 stimulates increased renal elimination of P. In this study, HOMA-IR positively correlates with P. This finding dictates that the rise of FGF23 that occurs in response to P retention (among other factors) is the risk factor of increased HOMA-IR instead. The association of FGF23 and IR could be related to the role of FGF 23 in chronic inflammatory state encountered in CKD patients. Singh et al. demonstrated that FGF23 stimulates the hepatic secretion of the inflammatory markers IL-6 and C-reactive protein. This finding demonstrates the contribution of FGF23 to the chronic inflammatory status of CKD patients [26].

To our knowledge, this study is the first to evaluate IR in non-diabetic normotensive CKD patients. The disclosure of serum P and FGF23, beside the other factors detected by multivariate regression, as risk factors of increased IR might add a new explanation for the strong association between these risk factors and the increased cardiovascular and overall mortality among CKD patients.

## Abbreviations

25 (OH) vit D: serum 25 hydroxy vitamin D
ACR: urine albumin/creatinine ratio
BMI: body mass index
c-terminal: carboxyl terminal
Ca: serum calcium
CKD: chronic kidney disease
eGFR: estimated glomerular filtration rate
FBG: fasting blood glucose
FBI: fasting blood insulin
FGF23: fibroblast growth factor-23
HOMA-IR: homeostatic model assessment of insulin resistance
HPLC: high performance liquid chromatography
IR: insulin resistance
MBD: mineral bone disease
P: serum phosphorus
PTH: serum parathormone
UA: serum uric acid
